# Pch2 Prevents Mec1/Tel1-Mediated Hop1 Phosphorylation Occurring Independently of Red1 in Budding Yeast Meiosis

**DOI:** 10.1371/journal.pone.0085687

**Published:** 2014-01-21

**Authors:** Yu-Hui Lo, Chi-Ning Chuang, Ting-Fang Wang

**Affiliations:** 1 Institute of Life Science, National Defense Medical Center, Taipei, Taiwan; 2 Institute of Molecular Biology, Academia Sinica, Taipei, Taiwan; National Cancer Institute, United States of America

## Abstract

A prominent feature of meiosis in most sexually reproducing organisms is interhomolog recombination whereby a significant fraction of the programmed meiotic double-strand breaks are repaired using intact homologous non-sister chromatids rather than sister chromatids. Budding yeast DNA damage checkpoint kinases Mec1 and Tel1 act together with the axial element protein Red1 to promote interhomolog recombination by phosphorylating another axial element protein Hop1. Mec1 and Tel1 also phosphorylate γH2A and the synaptonemal complex protein Zip1 independently of Red1 to facilitate premeiotic DNA replication and to destabilize homology-independent centromere pairing, respectively. It has been unclear why Hop1 phosphorylation is Red1-dependent. Here, we report that the pachytene checkpoint protein 2 (Pch2) specifically prevents Red1-independent Hop1 phosphorylation. Our findings reveal a new function for Pch2 in linking two axial element proteins Red1 and Hop1 thus coordinating their effects in meiotic recombination and the checkpoint network.

## Introduction

Budding yeast Mec1 and Tel1 are homologs of mammalian protein kinases ATR and ATM. These evolutionarily conserved signal transduction protein kinases are chromosome-associated and act as master regulators of the checkpoint responses to DNA double strand breaks (DSBs). Failure to repair DSBs can lead to mutations, chromosome rearrangement and genomic instability [1]. Mec1/ATR and Tel1/ATM preferentially phosphorylate their substrates at the SQ/TQ motifs, i.e., serine (S) and threonine (T) residues that precede glutamine residues. For example, γH2A (i.e., H2A at S129) phosphorylation occurs during vegetative growth S-phase and marks specific chromosomal domains that trigger DNA damage responses [2]. Several DNA repair and DNA damage checkpoint proteins are also phosphorylated by these two kinases during the vegetative growth cell cycle, e.g., Rad9, Rad52, RPA, Sae2 and Mrc1 [3], [4].

Mec1 and Tel1 also play important roles in meiosis, a specialized cell cycle in sexually reproductive organisms that produces haploid gametes or ascospores (the sexual spores in fungal ascomycetes). The central steps of meiosis in many organisms are the pairing and DNA recombination of homologous chromosomes (i.e., the parental chromosomes, each containing two sister chromatids) during the leptotene-zygotene transition. In many organisms, homologous chromosomes align (synapsis) together in the pachytene stage [5]. Meiotic recombination is initiated by Spo11-induced double strand breaks (DSBs), and chromosome synapsis is mediated by a tripartite structure named the synaptonemal complex (SC). The SC is a zipper-like protein complex that consists of a central element and two dense lateral/axial elements. The major structural component of budding yeast central element is Zip1 [6]. Zip1 also mediates nonhomologous centromere coupling (NHCC) during early meiosis [7]. The major structural components of the axial elements are the sister chromatid cohesion complex (Rec8/Scc3/Smc1/Smc3) [8] and three meiosis-specific components Hop1, Red1 and Mek1 [9], [10], [11]. Red1 and Hop1 prominently load to meiotic chromosomes just before SC assembly [12] or even before DSB formation [13]. Mek1 is a meiosis-specific protein kinase that upholds interhomolog bias during meiotic recombination [14], [15], [16], [17], [18], [19]. The meiotic checkpoint network detects a variety of ongoing meiotic cell-cycle events and relays this information to other (metabolically independent) processes, and eventually acts as a surveillance mechanism to halt cell-cycle progression and activate repair responses when necessary. Mec1/Tel1-mediated H2A-S129 phosphorylation appears at the onset of premeiotic S phase to trigger DNA damage responses, and this phosphorylation occurs independently of Spo11 or SC [20]. Upon meiotic DSB formation, Mec1 and Tel1 phosphorylate Zip1 at S75 [21], [22] to dynamically destabilize NHCC [21]. Rec114, an essential accessory factor of Spo11, is also downregulated by Mec1/Tel1-mediated phosphorylation to maintain genetically determined levels of DSBs [23]. Finally, Mec1 and Tel1 affect Hop1 activities (e.g., interhomolog recombination and SC assembly) by phosphorylation which occurs most profoundly at T318, because Hop1-T318 phosphorylation is required for chromosomal recruitment and activation of Mek1 [22], [24]. The Mek1 kinase phosphorylates multiple targets in meiosis, including T132 of Rad54 [17], a dsDNA-dependent ATPase required for Rad51 recombinase activity [25]. The forkhead-associated (FHA) domain of Mek1 is involved in positive feedback activity to stabilize Hop1-T318 phosphorylation against the dephosphorylation mediated by protein phosphatase 4 (PP4) [22].

Unlike DSB-dependent Zip1-S75 phosphorylation or DSB-independent H2A-S129 phosphorylation, DSB-dependent Hop1-T318 phosphorylation also requires the axial element protein Red1 [20]. Notably, Hop1 can bind to naked DNA *in vitro* [26] and to yeast meiotic chromosomes independently of Red1 [27]. Up until now it has been unclear why Red1 is specifically required for Hop1 or Hop1-T318 phosphorylation. In the present study, we report that deletion of the pachytene checkpoint protein 2 gene (*PCH2*) resulted in Red1-independent Hop1 phosphorylation during meiosis. Additional mutant analyses were carried out here to reveal the mechanism underlying this inhibitory function of Pch2.

## Materials and Methods

### Yeast strains, sporulation, and western blot analysis

All meiotic experiments were performed using diploid cells from the SK1 strain background. Spore viability was determined by tetrad dissection. The strain genotypes are given in Table 1.

**Table 1:** Spore Viability of Four-Spore Tetrads.

Strain	Genotype	% of viable spores	# of spores
WHY3285	*ho::hisG/″, leu2::hisG/″, HIS4::LEU2-*(*BamHI*)*/his4-X::LEU2-*(*BamHI*)*–URA3*	99	240
WHY6082	WHY3285, *pch2*Δ*::KanMX4/″*	84	216
WHY6448	WHY3285, *red1*Δ*::KanMX4/″*	0	216
WHY10491	WHY3285, *pch2*Δ*::KanMX4/″, red1*Δ*::KanMX4/″*	<1	288
WHY10532	WHY3285, *XRS2-13myc::KanMX4/″*, *red1*Δ*::KanMX4/″*	0	216
WHY10533	WHY3285, *xrs2*Δ*N-13myc::KanMX4/″*, *red1*Δ*::KanMX4/″*	0	216
WHY10535	WHY3285, *red1*Δ*::KanMX4/″, dot1*Δ*::KanMX4/″*	<1	220
WHY9523	WHY3285, *red1*Δ*::KanMX4/″, rec8*Δ*::leu2/″*	0	144
WHY9174	WHY3285, *pph3*Δ*::KanMX4/″*	51	216
WHY10541	WHY3285, *pph3*Δ*::KanMX4/″, red1*Δ*::KanMX4/″*	<1	216

Spore viabilities were determined following sporulation in liquid medium at 30°C.

Western blot analyses were carried out as recently described using primary antisera against Hsp104 (1∶10,000 dilution), Hop1 (1∶10,000 dilution), phosphorylated Hop1-T318 (1∶10,000 dilution), phosphorylated Zip1-S75 (1∶10,000 dilution), phosphorylated Rad54-T132 (1∶1000 dilution) and phosphorylated H2A-S129 (1∶50,000 dilution; Millipore, MA, USA) [20], [22]. After hybridization with the primary antisera, the blots were incubated with the secondary goat anti–rabbit IgG (10,000 dilution) or goat anti–guinea pig IgG (50,000 dilution; phosphorylated Zip1-S75) conjugated with alkaline phosphatase (Jackson ImmunoResearch Laboratories). The protein bands were visualized using the ChemiLucent ECL Detection System (Millipore, Billerica, MA, USA) and imaged by exposure to X-ray film. All experiments were repeated twice, and the results of representative sporulation time courses are shown.

The relative intensities of protein bands of interest from X-ray films were obtained with a Biospectrum 600 imaging system (UVP, Upland, CA, USA) containing an OptiCam 600 camera (Canon, Japan). For quantification, protein bands were plotted on a bar graph using VisionWorksLS Image Acquisition and Analysis Software (UVP, Upland, CA, USA). The intensities of phosphorylated proteins and of the loading control (Hsp104) were acquired from X-ray films by subtracting an equally sized background, respectively. For normalization, intensity of phosphorylated protein was divided by that of the loading control at the indicated time point [20], [22].

## Results

### Pch2 prevents Red1-independent Hop1-T318 phosphorylation

Budding yeast Pch2 was originally proposed to act in concert with Zip1 and the SC initiation protein Zip3 to detect aberrant or incomplete SC intermediates as part of the pachytene checkpoint machinery [28], [29], [30]. The majority of the Pch2 protein localizes to the nucleolus, where Pch2 prevents meiotic interhomolog recombination in the ribosomal DNA by excluding Hop1 [28]. Later, it was reported that Zip1 and Hop1 exhibited a distinct and often complementary staining pattern along wild-type (WT) pachytene chromosomes, with the same tendency for Zip1 and Red1. In contrast, in the *pch2*Δ mutant, both Hop1 and Zip1 were promiscuously or uniformly loaded along pachytene chromosomes [30]. Because Pch2 can regulate the localization of Hop1 and Zip1 along WT pachytene chromosomes, we speculated that Pch2 might have a role in regulating Hop1 phosphorylation and/or Zip1 phosphorylation.

**Figure 1:**
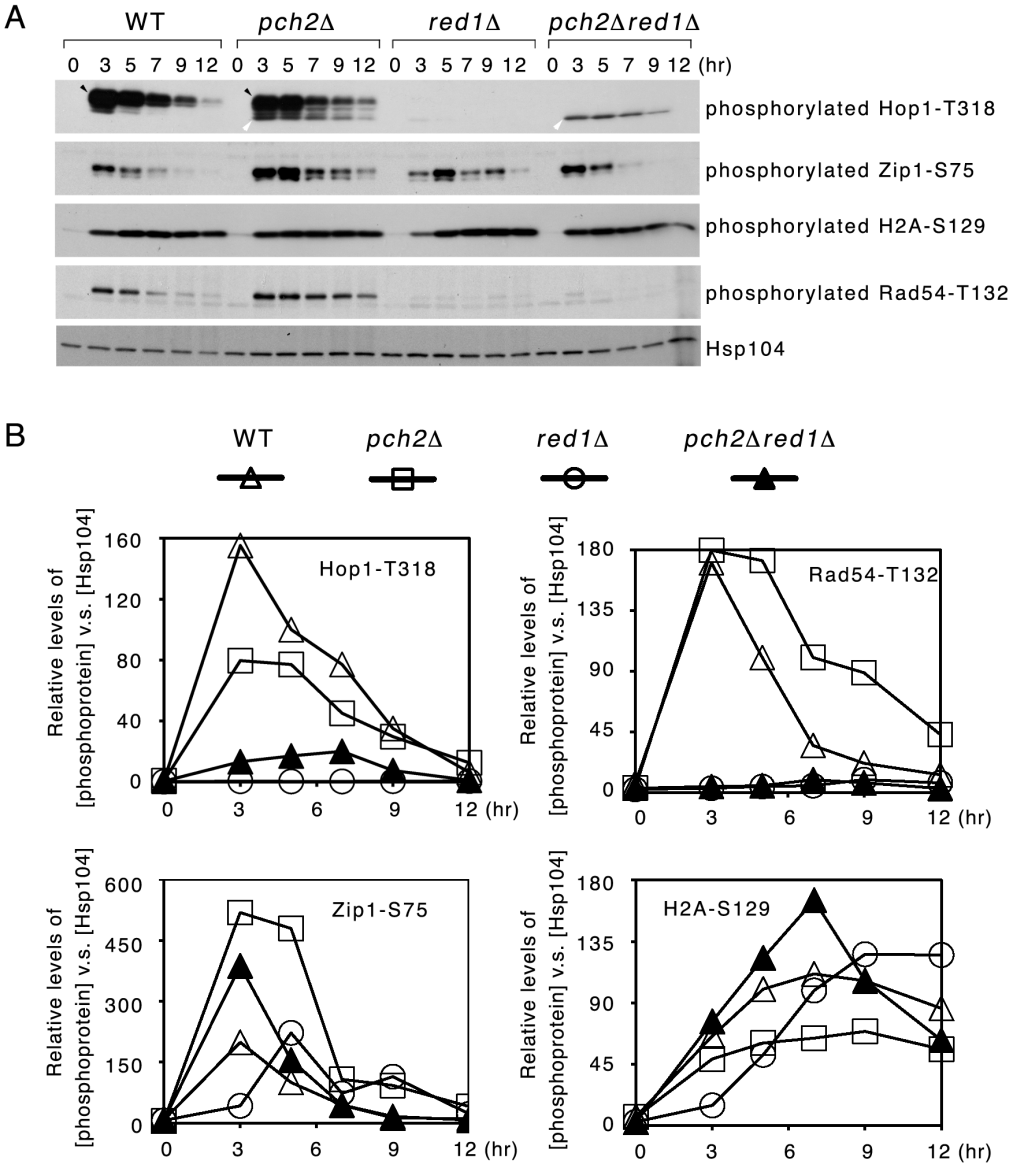
*PCH2* specifically prevents Mec1/Tel1-mediated Hop1 hypophosphorylation in WT and *red1*Δ. (A) Western blot time-course analyses of phosphorylated Hop1-T318, Zip1-S75, H2A-S129 and Rad54-T132 in sporulating cells were performed as recently described [20], [22]. Hsp104 was used as a loading control. All experiments were repeated twice, and the results of representative sporulation time courses are shown. Hyperphosphorylated Hop1 and hypophosphorylated Hop1 are indicated with black and white arrows, respectively. (B) Quantification of phosphorylated protein in (A) (see Materials & Methods). Relative levels of phosphorylated proteins were determined by setting the level of WT “5 hr” as 1.

All yeast strains were induced to undergo relatively synchronous sporulation. At the indicated time points, cells were harvested for preparation of total cell lysates according to the TCA precipitation protocol described previously [20], [27], [31]. Western blot time-course analyses with antisera against phosphorylated Hop1-T318 (Figure 1, top panel) revealed that Hop1 was hyperphosphorylated (indicated with black arrow) at 3 h after WT cells entered meiosis, and the hyperphosphorylated Hop1 gradually diminished at later meiotic time points. In *pch2*Δ, Hop1 proteins appeared to be both hyperphosphorylated (black arrow) and hypophosphorylated (white arrow) at 3 h time points. Hypophorylated Hop1 migrated faster than hyperphosphorylated Hop1 in the SDS-PAGE gel (Figure 1). Next, we confirmed that Hop1-T318 was hardly phosphorylated in *red1*Δ as reported before [20], [22], [24] (Figure 1). Remarkably, deletion of the *PCH2* gene in *red1*Δ induced hypophosphorylated Hop1 (white arrow) but not hyperphosphorylated Hop1. In contrast, the expression patterns of phosphorylated Zip1-S75 and phosphorylated H2A-S129 were only slightly different in the four strains examined here. These results indicate that Pch2 has a specific function in preventing Hop1 undergoing peculiar “hypophorylation” in both WT and *red1*Δ.

Hop1-T318 phosphorylation is required for Mek1 activation to phosphorylate Rad54-T132 [22]. We found that Mek1-mediated Rad54-T132 phosphorylation did not occur in *red1*Δ *pch2*Δ, mirroring the result in *red1*Δ (Figure 1, second bottom panel). The *red1*Δ *pch2*Δ mutant, again mirroring *red1*Δ, generated very few or no viable spores (Table 1). Accordingly, the hypophosphorylated Hop1 protein observed in *red1*Δ *pch2*Δ was defective in Mek1 activation and interhomolog recombination.

### Hop1-T318 is not phosphorylated in *red1*Δ *rec8*Δ

**Figure 2:**
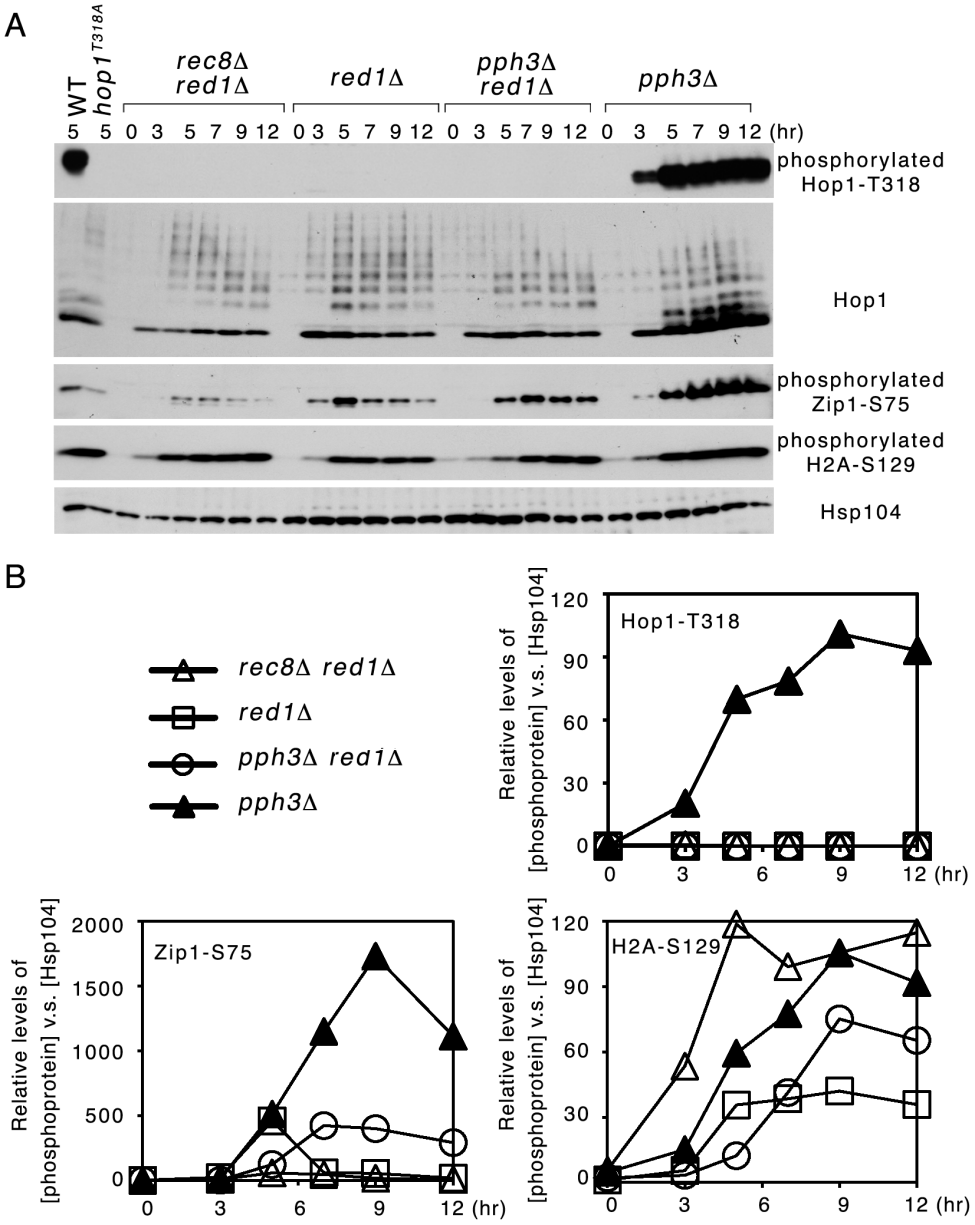
Rec8 and the catalytic subunit of PP4, Pph3, do not affect Red1-independent Hop1 phosphorylation. (A) Western blot time-course analyses were performed as described in Figure 1. To validate the specificity of antisera against phosphorylated Hop1-T318, total meitoic cell lysates from strains carrying the wild-type (WT) *HOP1* allele (at 5 hr) and the *hop1^T318A^* mutant allele (at 5 hr) were used as positive and negative controls, respectively. The *hop1^T318A^* variant encodes a mutant protein in which the T318 residue of Hop1 has been mutated to alanine. (B) Quantification of phosphorylated protein in (A). Relative levels of phosphorylated proteins were determined by setting the level of WT “5 hr” as 1.

Red1 and the meiosis-specific cohesin Rec8 are both required for normal sister chromatid cohesion [8], [32], [33] and normal DSB levels [15]. Unlike *red1*Δ, the *red1*Δ *rec8*Δ double mutant is able to establish homolog bias [15]. Rec8 functions in cohesion rather than axis integrity, preventing nonspecific chromosome interactions, as deletion of the cohesin subunit Rec8, but not Red1 or Hop1, caused an increase in homolog-nonspecific chromosome interaction [34]. Hop1-T318 phosphorylation is required for interhomolog recombination and viable spore generation in the WT [22], [24], but whether Hop1 or Hop1-T318 is phosphorylated in the *red1*Δ *rec8*Δ double mutant is unknown. This study demonstrated that the *red1*Δ *rec8*Δ double mutant, like *red1*Δ, exhibited no Hop1-T318 phosphorylation (Figure 2), indicating that Hop1-T318 phosphorylation is not required to establish interhomolog recombination in *red1*Δ *rec8*Δ. Notably, the *red1*Δ *rec8*Δ double mutant cells generated much fewer tetrads (<5%) than the WT cells, and these tetrads hardly contained any viable spores (<1%; Table 1), probably due to increases in homolog-nonspecific chromosome interactions in the absence of Rec8 [34].

Our results here also suggest that Rec8 does not affect Pch2 in repressing Red1-independent Hop1-T318 phosphorylation. Finally, the *red1*Δ *rec8*Δ double mutant exhibited lower levels of DSB-dependent Zip1-S75 phosphorylation than *red1*Δ (Figure 2), consistent with previous results that indicated lower DSB levels in *red1*Δ *rec8*Δ than in *red1*Δ [15]. In contrast, the steady-state levels of DSB-independent H2A-S129 phosphorylation were higher in *red1*Δ *rec8*Δ than in *red1*Δ or *red1*Δ *pph3*Δ (Figure 2B).

### Pch2 does not use PP4 for Hop1 dephosphorylation

PP4 dephosphorylates several targets of Mec1 and Tel1 kinases (e.g., H2A-S129, Zip1-S75 and Hop1-T318) during budding yeast meiosis [21], [22]. Therefore, next we examined whether Pch2 might function to promote PP4-mediated Hop1-T318 dephosphorylation. We found that Hop1-T318 was not phosphorylated in *red1*Δ *pph3*Δ during meiosis. Pph3 is the catalytic subunit of PP4 [35], [36]. The overall order of steady-state levels of Hop1-T318 phosphorylation was *pph3*Δ >> *red1*Δ *pph3*Δ ∼ *red1*Δ. Therefore, the inhibition of Hop1 phosphorylation by Pch2 apparently occurs prior to PP4. Notably, PP4 still could dephosphorylate Zip1-S75 and H2A-S129 in the absence of *RED1*, as the steady-state levels of DSB-dependent Zip1-S75 phosphorylation and DSB-independent H2A-S129 phosphorylation were higher in *red1*Δ *pph3*Δ than in *red1*Δ (Figure 2).

### Neither *dot1*Δ nor *xrs2*Δ*N* can recapitulate the effects of *pch2*Δ in prevention of Red1-independent Hop1-T318 phosphorylation

The nucleolar localization of Pch2 depends on two silencing factors, Sir2 and Dot1 (also known as Pch1) [28], [37]. Sir2 and Dot1, like Pch2, are dispensable during WT meiosis, but they are essential to prevent progression of meiosis in the absence of Zip1 [28], [37]. The Dot1 protein methylates Lys79 of histone H3 [38]. Recently, it was proposed that Dot1-mediated histone H3K79 methylation controls Hop1 localization by excluding Pch2 from the chromosomes, thus driving localization of Hop1 along the chromosome axes and enabling full Mek1 activation [39]. Pch2 also physically interacts with the N-terminal domain of Xrs2, a component of the Mre11-Rad50-Xrs2 complex, that acts as the site of unprocessed DSBs. An N-terminal deletion (*xrs2*Δ*N*) that deletes the first 313 amino acid coding region of *XRS2* recapitulates the *pch2*Δ phenotype for signaling unresected DSBs to delay meiotic cell cycle progression [40].

**Figure 3:**
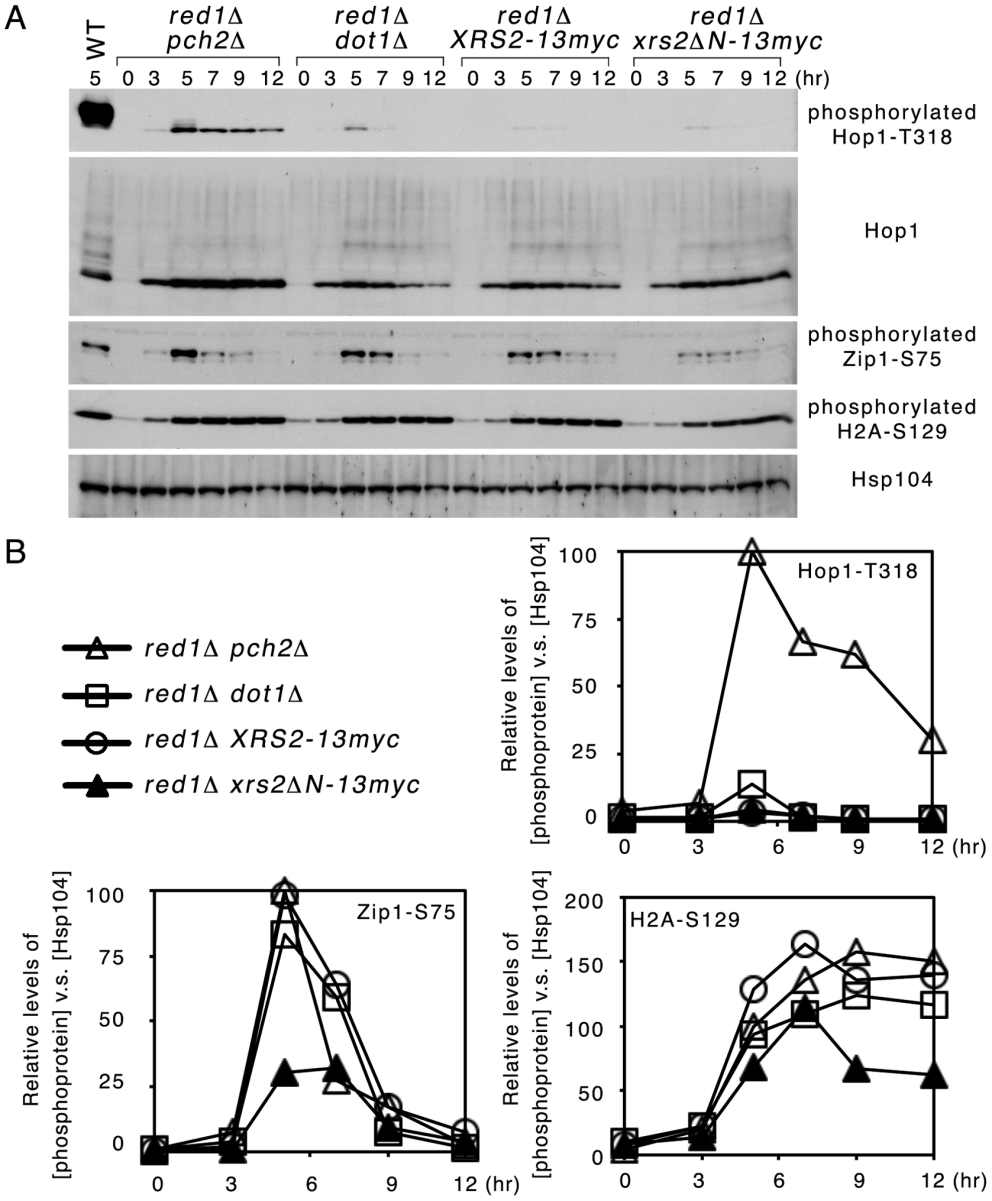
Neither *dot1*Δ nor *xrs2*Δ*N* can recapitulate the effects of *pch2*Δ on preventing Red1-independent Hop1-T318 phosphorylation. (A) Western blot time-course analyses were performed as described in Figure 1. (B) Quantification of phosphorylated protein in (A). Relative levels of phosphorylated proteins were determined by setting the level of *red1*Δ *pch2*Δ “5 hr” as 1.

Next, we examined if *dot1*Δ or *xrs2*Δ*N* also affected Red1-independent Hop1 phosphorylation. The order of steady-state levels of Hop1-T318 phosphorylation was *red1*Δ *pch2*Δ > *red1*Δ *dot1*Δ > *red1*Δ *xrs2*Δ*N* ∼ *red1*Δ (Figure 3). As in *red1*Δ *pch2*Δ and *red1*Δ, both *red1*Δ *dot1*Δ and *red1*Δ *xrs2*Δ*N* double mutants generated very few or no viable spores (Table 1). Our results also revealed that neither *xrs2*Δ*N* nor *dot1*Δ significantly affected DSB-dependent Zip1-S75 phosphorylation or DSB-independent H2A-S129 phosphorylation (Figure 2), consistent with a previous report that the *xrs2*Δ*N* mutant is dispensable for early meiotic events, including DNA replication, the formation of normal levels of DSBs or crossover recombination [40], [41]. We conclude that Pch2 functions independently of Xrs2 and Dot1 in preventing Red1-independent Hop1 phosphorylation.

## Discussion

Our study clearly demonstrates that Pch2 has a specific role in regulating the interdependence of Red1 and Hop1. The *pch2*Δ deletion specifically induced DSB-dependent Hop1 phosphorylation in *red1*Δ but only slightly affected DSB-dependent Zip1-S75 phosphorylation (Figure 1). Therefore, this new function of Pch2 is distinct from those of Pch2 in regulating global levels of Spo11-induced DSBs [42], [43]. We also showed that the role of Pch2 in preventing Red1-independent Hop1 phosphorylation apparently occurred independently of Rec8, PP4 (Figure 2), Xrs2 and Dot1 (Figure 3). Rec8, a meiosis-specific cohesin [8], is required for normal levels of DSBs [15]. PP4 mediates Hop1 dephosphorylation [22]. Dot1-meidated histone H3K79 methylation controls Hop1 localization by excluding Pch2 from the chromosomes (including the nucleous) [39]. Xrs2 physically links Pch2 to Tel1, thus activating Tel1 for Hop1 phosphorylation in response to unresected DSBs [40]. Taken together, our results indicate that the inhibitory function of Pch2 in preventing Red1-independent Hop1 phosphorylation likely occurs before DSB formation or at least prior to Tel1 activation, which occurs at sites of unresected DSBs. Because Pch2 has a role in regulating the normal chromosomal localization of Hop1 [28], [30], [39] and the proteins necessary for DSB formation (i.e., pre-DSB recombination complexes) preferentially reside on Hop1- and Red1-rich chromosomal regions [13], we inferred that Pch2 might act together with Red1 or the Red1 containing chromosomal scaffolds to exclude Hop1 from promiscuous loading to those DSBs sites far away from the Red1-rich chromosome axis, thus preventing Red1-independent Hop1 phosphorylation. In the future, we will further investigate whether and how Red1 or its interacting proteins affect Pch2 in regulating Hop1 in association with meiotic chromosomes *in vivo* and/or naked DNA *in vitro*. It will also be of interest to learn why the hypophorylated Hop1-T318 in *red1*Δ *pch2*Δ is incapable of activating Mek1.

Finally, there are some interesting features of the 3 SQ/TQ phosphoproteins in this study, particularly timing differences. The steady-state levels of these SQ/TQ phosphoproteins can be affected by multiple factors, including (i) overall DSB levels; (ii) activation of Mec1 and Tel1; (iii) protein phosphatases (e.g., PP4); (iv) Proteins which associate with the phosphorylated SQ/TQ motifs. A typical example is that Mek1 can physically stabilize phosphorylated Hop1-T318 against PP4-mediated dephosphorylation [22]. It is unclear if there is any protein can recognize and stabilize phosphorylated Zip1-S75 or phosphorylated H2A-S129 in meiosis; (v) proteins which regulate chromosomal or subcellular localization of the SQ/TQ phosphoproteins, e.g., Pch2 v.s. phosphorylated Hop1-T318 (Figure 1–3); (vi) proteins which regulate degradation of the SQ/TQ phosphoproteins. In the long run, the timing differences will be a complex problem to be addressed.
